# HIV Tat-Mediated Induction of Monocyte Transmigration Across the Blood–Brain Barrier: Role of Chemokine Receptor CXCR3

**DOI:** 10.3389/fcell.2021.724970

**Published:** 2021-08-30

**Authors:** Fang Niu, Ke Liao, Guoku Hu, Shamsudheen Moidunny, Sabita Roy, Shilpa Buch

**Affiliations:** ^1^Department of Pharmacology and Experimental Neuroscience, University of Nebraska Medical Center, Omaha, NE, United States; ^2^Division of Clinical Research and Evaluative Sciences, Department of Medicine, Creighton University, Omaha, NE, United States; ^3^Smidt Heart Institute, Cedars-Sinai Medical Center, Los Angeles, CA, United States; ^4^Department of Surgery, Sylvester Comprehensive Cancer Center, Miller School of Medicine, University of Miami, Miami, FL, United States

**Keywords:** monocytes, CXCR3, HIV Tat, BBB, transmigration, neuroinflammation

## Abstract

HIV trans-activator of transcription (Tat), one of the cytotoxic proteins secreted from HIV-infected cells, is also known to facilitate chemokine-mediated transmigration of monocytes into the brain leading, in turn, to neuroinflammation and thereby contributing to the development of HIV-associated neurocognitive disorders (HAND). The mechanism(s) underlying HIV Tat-mediated enhancement of monocyte transmigration, however, remain largely unknown. CXC chemokine receptor 3 (CXCR3) that is expressed by the peripheral monocytes is known to play a role in the monocyte influx and accumulation. In the present study, we demonstrate for the first time that exposure of human monocytes to HIV Tat protein resulted in upregulated expression of CXCR3 leading, in turn, to increased monocyte transmigration across the blood–brain barrier (BBB) both in the *in vitro* and *in vivo* model systems. This process involved activation of toll-like receptor 4 (TLR4), with downstream phosphorylation and activation of TANK-binding kinase 1 (TBK1), and subsequent phosphorylation and nuclear translocation of interferon regulatory factor 3 (IRF3), ultimately leading to enhanced expression of CXCR3 in human monocytes. These findings imply a novel molecular mechanism underlying HIV Tat-mediated increase of monocyte transmigration across the BBB, while also implicating a novel role of CXCR3-dependent monocyte transmigration in HIV Tat-mediated neuroinflammation.

## Introduction

Human Immunodeficiency Virus-1 (HIV-1) continues to be a major public health concern, despite the advent of combination antiretroviral therapy (cART) (Clifford and Ances, 2013; Chen et al., 2014). While the effectiveness of cART in suppressing viremia has resulted in increased life spans of those infected with the virus, paradoxically however, long-term dependence on anti-retrovirals coupled with persistent low-level virus replication and presence of viral Tat protein in the CNS, leads to a neuroinflammatory milieu that underlies the development of HIV-associated Neurocognitive Disorders (HAND) (Guha et al., 2018; Hellmuth et al., 2018; Thangaraj et al., 2018).

Monocytes are a type of circulating leukocytes that are derived from the progenitors in the bone marrow and that have the potential to differentiate into macrophages following an inflammatory response (Honold and Nahrendorf, 2018). Monocyte-derived macrophages, however, have been found to accumulate in the brain parenchyma during the progression of neuroinflammatory and neurodegenerative diseases, including Alzheimer’s Disease (AD) (Hohsfield and Humpel, 2015), Amyotrophic Lateral Sclerosis (ALS) (Butovsky et al., 2012), as well as HAND (Eugenin et al., 2006; Williams et al., 2012). In HAND, it has been suggested that HIV-infected monocytes transmigrate into the brain and spread the virus to cells such as the microglia (Cenker et al., 2017), macrophages (Burdo et al., 2013) and pericytes (Bertrand et al., 2019). Virus-infected cells have been shown to continually release cytokines, chemokines, and neurotoxic viral proteins, all of which lead to chronic neuroinflammation in the CNS (Griffin, 1997; Tatro et al., 2014). Transmigration of HIV-infected monocytes into the brain has been shown to contribute to the pathogenesis of HAND (Eugenin et al., 2006; Bethel-Brown et al., 2012).

Chemokines and their receptors orchestrate monocyte transmigration and recruitment into tissues. There are reports of increased expression of chemokines such as CCL2 and CXCL10 in the plasma and cerebrospinal fluids of HIV-infected patients with neurocognitive impairment compared with asymptomatic HIV-infected individuals (Yuan et al., 2015), thus underscoring the role of monocyte recruitment in the progression of neurologic complications of HIV-1. The critical role and mechanism(s) of action of CCL2 (also known as monocyte chemotactic protein-1, MCP-1)-mediated monocyte transmigration across the BBB have been extensively studied (Eugenin et al., 2006; Dhillon et al., 2008). The role of CXCL10-mediated monocyte transmigration in HAND, however, has received less attention. CXCL10 (IFN-γ -inducible protein 10, also known as IP-10), a CXC chemokine, was one of the chemokines found to be significantly elevated during the early stages of HIV-1 infection (Jiao et al., 2012). In untreated HIV-infected individuals levels of CXCL10 were shown to be significantly increased compared with uninfected individuals, and furthermore, antiretroviral therapy (ART) failed to downregulate the expression of CXCL10 (Kamat et al., 2012; Castley et al., 2016). HIV-infected monocyte transmigration into the CNS has been demonstrated during the early stages of HIV infection (Williams et al., 2014), implying thereby a role of CXCL10 in monocyte transmigration into the brain.

C-X-C motif chemokine receptor 3 (CXCR3), a G protein-coupled receptor of CXCL10, is shown to be commonly expressed on monocytes, Th1 T cells and microglia (Muller et al., 2007; Tokunaga et al., 2018). CXCR3/CXCL10 interactions drive leukocyte transmigration (Butler et al., 2017). It has been shown that CXCR3 is present on a small subset of peripheral blood monocytes and a higher percentage of monocytes recruited to the inflammatory sites in homeostatic arterial remodeling triggered by hemodynamic stress in mice (Zhou et al., 2010). The role of CXCR3 in monocyte/macrophage transmigration into the CNS in HAND remains to be explored.

HIV Tat protein that is secreted by the virus-infected cells and is a known virotoxin, and plays a key role in HIV-associated neurodegeneration owing to its effects on neurotoxicity and neuroinflammation (Niu et al., 2014; Thangaraj et al., 2018; Hijmans et al., 2019; Santerre et al., 2019). For example, it has been demonstrated that viral HIV Tat-mediated monocyte transmigration across the BBB involved monocyte adhesion via upregulation of the expression of adhesion proteins ICAM-1 and VCAM-1 (Song et al., 2007). Furthermore, HIV Tat was also found to promote monocyte infiltration by inducing inflammatory mediators in the CNS (Pu et al., 2003) and by upregulating the expression of CCR5 on monocytes (Weiss et al., 1999). Moreover, Ben Haij et al. (2013) showed that HIV Tat protein directly binds to toll-like receptor 4 (TLR4) which plays a crucial role in inflammation. The aim of the present study was to identify the role of CXCR3 in HIV Tat-mediated monocyte transmigration and the involvement of TLRs in HIV Tat induced upregulated expression of CXCR3 in human monocytes. Understanding the role of CXCR3 in HIV Tat-mediated monocyte transmigration could provide insights into the future development of therapeutic targets aimed at abrogating neuroinflammation associated with HAND.

## Materials and Methods

### Animals

C57BL/6 mice (male, 6–8 weeks) were purchased from Charles River Laboratories, Inc., CX3CR1-GFP homozygous mice were obtained from the Jackson Laboratory (Bar Harbor, ME, United States). These mice have a targeted deletion of CX3CR1 that is replaced by a GFP reporter insertion. All animals were housed under conditions of constant temperature and humidity on a 12-h light/12-h dark cycle, with lights on at 7:00 AM. Food and water were available *ad libitum*. All animal procedures were performed according to the protocols approved by the Institutional Animal Care and Use Committee at the University of Nebraska Medical Center.

### Macaques

Sections of archival macaque brain tissues collected from our previous study (Bokhari et al., 2011) were used in this study. Briefly, six 2- to 3-year-old Indian rhesus macaques (*Macaca mulatta*) were purchased from the Caribbean Research Primate Center. The animals were randomly divided into two groups: Control (*n* = 4), acute SIV (*n* = 6) and chronic SIV (*n* = 3). The animals were inoculated intravenously with approximately 10^4^ plaque-forming units of SIV R17/71E for 3 weeks (Acute) or 12 months (Chronic). Details of the infection and viral loads have been reported earlier (Bokhari et al., 2011).

### Cell Culture

Primary Human Brain Microvascular Endothelial Cells (HBMECs) were obtained from Dr. Monique Stins (Johns Hopkins University). Cells were cultured in RPMI 1640 medium (GE Healthcare Life Sciences, Pittsburgh, PA, United States) containing 10% heat-inactivated fetal bovine serum (Atlanta Biologicals, Flowery Branch, GA, United States), 10% Nu-Serum (BD Biosciences, San Jose, CA, United States), 2 mM L-glutamine (Invitrogen, Carlsbad, CA, United States), 1 mM pyruvate (GE Healthcare Life Sciences), penicillin (100 units/ml) and streptomycin (100 μg/ml, Gibco), essential amino acids (HyClone), and MEM vitamin solution (HyClone). Purified HBMECs were positive for endothelial makers DiI-AcLDL, ZO-1 and β-catenin and were found to be >99% pure after exclusion of staining for non-endothelial cell type markers (GFAP, smooth muscle actin, cytokeratin, and macrophage antigens) as described previously (Wen et al., 2011).

### Monocyte Isolation

Human monocytes were purchased from the Elutriation Core Facility in the Department of Pharmacology and Experimental Neuroscience at UNMC which yield >97% purity. Monocytes were obtained from HIV-1, HIV-2, and hepatitis B seronegative donor leukopacks and separated by countercurrent centrifugal elutriation as previously described (Gendelman et al., 1988). Freshly elutriated monocytes were cultured in DMEM (Gibco) containing 10% heat-inactivated human serum (Thermo Fisher Scientific, Hudson, NH, United States), 2 mM L-glutamine (Invitrogen), 100 mg/ml gentamicin and 10 mg/ml ciprofloxacin (Sigma-Aldrich, St. Louis, MO, United States).

### Isolation and Cultivation of Bone Marrow-Derived Monocytes (BMM)

CX3CR1-GFP homozygous mice (Jackson Laboratory), 6–8 weeks of age, were used as BMM donors as previously described (Niu et al., 2019). Briefly, the femur of CX3CR1-GFP mice was removed, and bone marrow cells were dissociated into single-cell suspensions that were supplemented with 1,000 U/ml of macrophage colony-stimulating factor and cultured for 5 days.

### Reagents

Full length (101 amino acids) recombinant HIV-1 MN Tat was produced in the *Escherichia coli* expression system and purchased from ImmunoDiagnostics (Catalog. 1032; Woburn, MA, United States). CU-CPT22, OxPAPC, LPS, and Amlexanox were purchased from Sigma-Aldrich. CXCR3 antagonist AMG487 was obtained from Tocris Bioscience (Bristol, United Kingdom). The concentrations of these inhibitors were based on the concentration curve study and our previous reports (Niu et al., 2019). Cell Tracker Green CMFD was purchased from Invitrogen. Mouse CXCL10 Recombinant Protein was purchased from Invitrogen. Human CXCL10 Recombinant Protein and human CXCL10/IP-10 DuoSet Kit were obtained from R&D Systems (Minneapolis, MN, United States).

### Proteome Profiler Array

Human Cytokine Array Panel A was purchased from R&D Systems (Minneapolis, MN, United States). Frozen postmortem brain samples of the frontal cerebral cortex from HIV-1 infected patients (*n* = 3) or negative controls (*n* = 3) were obtained from the UNMC PEN Brain Banking for Study of Neurologic Diseases (IRB: 056–00-FB). The clinical characteristics of the patients included in this study were described previously (Hu et al., 2019). Brain tissues were homogenized in RIPA buffer and equal concentrations (1 μg/μl) of samples were used to probe the cytokine array membranes according to the manufacturer’s instructions. The chemiluminescent signal from the bound cytokines present in the brain lysates was detected by the FluorChem R system (ProteinSimple, CA, United States).

### ELISA

Frozen postmortem brain samples of frontal cortices from HIV-1 infected individuals (*n* = 4) or negative controls (*n* = 4) were obtained from the UNMC PEN Brain Banking for Study of Neurologic Diseases (IRB: 056–00-FB). The clinical characteristics of the patients included in this study were described previously (Hu et al., 2019). Brain tissues were homogenized in RIPA buffer and balanced to the concentration 1 μg/μl. 100 μl samples were assessed for expression of CXCL10 protein using a Human CXCL10/IP-10 DuoSet Kit (R&D Systems) according to the manufacturer’s instructions. Samples were assessed for CXCL10 expression.

### Immunofluorescence Staining

Cultured human monocytes were fixed with 4% formaldehyde in PBS for 20 min at RT. The cells were washed three times with PBS, permeabilized with 0.3% Triton X-100 for 30 min, and blocked in 10% goat serum in PBS for 2 h at RT, followed by incubation with CXCR3 antibody (Santa Cruz, Cat# sc-137140, Dallas, TX, United States) at 1:200 dilution. The cells were washed with PBS and incubated with Alexa Fluor 488–conjugated anti-mouse immunoglobulin G (Invitrogen) for 1 h at RT. After the final wash with PBS, cells were mounted using the mounting medium (Prolong Gold Antifade Reagent; Invitrogen) on the slides. Fluorescent images were acquired at RT on a Zeiss Observer, using a Z1 inverted microscope with a 63×/1.4 oil-immersion objective. Images were processed with the AxioVs 40 Version 4.8.0.0 software (Zeiss). Photographs were acquired with an AxioCam MRm digital camera and were analyzed using the ImageJ software.

### Flow Cytometry

Cells were stained for flow cytometry according to a previously published protocol (Niu et al., 2019). Human monocytes were washed and resuspended in 1 ml of staining buffer (PBS with 2% FBS), following which the cells were counted and incubated with anti-CD16/CD32 (BD Biosciences, San Jose, CA, United States) (2.5 μg/10^6^ cells) to block FcγII/III receptors. Human CXCR3 PE-conjugated antibody (R&D, FAB160P, 10 μL/10^6^ cells) was added to the cells, and the mixture was incubated for 30 min in dark on ice. Following three washes with staining buffer, cells were fixed with 0.5% PFA and analyzed on an LSR II flow cytometer (BD Biosciences) using the FACSDiva software.

### ChIP Assay

The ChIP assay was performed according to the manufacturer’s instructions (Upstate, Billerica, MA, United States) with slight modifications. Following treatment of cells, 18.5% fresh formaldehyde was added directly to the cell culture medium (final concentration 1%) and cells were incubated for additional 10 min at RT followed by quenching with 125 mM glycine. The cells were then washed using pre-chilled PBS containing 1× protease inhibitor mixture and pelleted by spinning at 800 g at 4°C. Cell pellet was resuspended in 200 μl SDS lysis buffer and was incubated for 10 min on ice. DNA was sheared by sonication and a total of 50 μl of the sheared cross-linked chromatin was then mixed with 20 μl protein A magnetic beads and 5 μg of respective antibodies -IRF3 (Santa Cruz, sc-33641), acetyl histone H3 (positive control), and normal rabbit IgG (negative control) in dilution buffer overnight at 4°C. The magnetic beads bound to the Ab-chromatin complex were subsequently washed with 0.5 ml each of a series of cold wash buffers in the order of low salt buffer, high salt buffer, LiCl buffer, and Tris–EDTA buffer. The crosslinking of protein-DNA complexes was then reversed to free the DNA by incubating the complex at 62°C for 2 h, followed by purification using the DNA purification spin columns according to the manufacturer’s instructions. Finally, the purified DNA was amplified (35 cycles) by PCR to identify the promoter region containing IRF3 binding site “ACTTAAAAGAAACTG.” The sequences of the primers used to identify the CXCR3 promoter bound to IRF3 were as follows: sense, TGGCATTCTGTAACTTGCTTCTTC and antisense, AAGGTGGGCGTGAGGATTG.

### Tissue Source and Immunofluorescence Staining

Formalin-fixed, paraffin-embedded sections of frontal cerebral cortex from HIV-1 infected individuals (*n* = 3) or negative controls (*n* = 3) were obtained from National NeuroAIDS Tissue Consortium (NNTC) (see Table 1 for clinical data) and stained with antibodies specific for CXCR3 (1:200; ab154845; Abcam) overnight at 4°C. Briefly, brain sections were washed three times in PBS and incubated with Alexa Fluor 488 AffiniPure Fab Fragment Goat Anti-Rabbit IgG (H+L) (Jackson ImmunoResearch Laboratories, 111-547-003, West Grove, PA, United States) for 45 min at RT, followed by three washes with PBS. Brain sections were then stained with CD68 (1:100; ab955; Abcam) and TMEM119 (1:100; ab185333; Abcam) antibodies overnight at 4°C. Macaque brain tissues were stained with antibodies specific for CD68 (1:50; M0814; Dako, Glostrup, Denmark) and CXCR3 (1:200; ab154845; Abcam) overnight at 4°C. All the brain sections were washed three times in PBS followed by incubation in Alexa Fluor 594-conjugated anti-mouse, Alexa Fluor 488-conjugated anti-rabbit, and Alexa Fluor 647-conjugated anti-rabbit immunoglobulin G (Invitrogen) for 2 h at RT. After the final wash with PBS, sections were mounted using the mounting medium and fluorescent images acquired at RT on a Zeiss Observer using a Z1 inverted microscope with a 40×/1.3 oil-immersion objective and Z-stacks generated from images taken at 2-μm intervals. Images were processed using the AxioVs 40 Version 4.8.0.0 software (Zeiss, Oberkochen, Germany). Photographs were acquired with an AxioCam MRm digital camera and were analyzed using the ImageJ software. For human sections, CD68+/TMEM119− cells in parenchyma were counted and the fluorescence intensity of CXCR3 in CD68+/TMEM119− cells was quantified. For macaque sections, CD68+ cells in parenchyma were counted and the fluorescence intensity of CXCR3 in CD68+ cells was quantified.

**TABLE 1:** Clinical characteristics of the patients included in this study.

Case number	HIV-1 infection	Age (years)	Gender	Neurocognitive assessment	Antiretroviral therapy
1 (7101958083)	Negative	46	Male	Unable to reliably assign neurocognitive diagnosis	N/A
2 (7102308387)	Negative	49	Male	Unable to reliably assign neurocognitive diagnosis	N/A
3 (3015)	Negative	64	Female	Neuropsychological impairment or dementia attributable to another cause	N/A
4 (4084)	HIV+	46	Male	Neuropsychological impairment or dementia attributable to another cause	No ARV
5 (4115)	HIV+	49	Male	Normal	HAART at last visit (KTA, TRU)
6 (4129)	HIV+	59	Female	Normal	HAART at last visit (TRU, RGV, isentress)

### Reverse Transcription and qRT-PCR

Reverse transcription and qRT-PCR were performed as described previously (Niu et al., 2014). Briefly, total RNA was extracted using the TRIzol reagent (Invitrogen) according to the manufacturer’s instructions. Quantitative analyses of mRNA were conducted using ABI 7500 Fast Real-Time PCR system (Applied Biosystems, Foster City, CA, United States). Real-time PCR amplifications were carried out for 40 cycles (denaturation: 30 s at 95°C; annealing: 1 min at 60°C) and RT-PCR for 28 cycles (denaturation: 30 s at 94°C; annealing: 30 s at 49°C; extension: 30 s at 72°C). The target mRNA expression levels were first normalized to the housekeeping gene β-actin. Then fold changes of mRNA expression levels were calculated by comparing to the control group.

Hu CXCR3, forward primer: 5′-CCTCTACAGCCTCCTCTT-3′; reverse primer: 5′-GCTCCTGCGTAGAAGTTG-3′. Hu TLR1, forward primer: 5′-TGGACTTCTGACATCTTATC-3′; reverse primer: 5′-CTTGAGGTTCACAGTAGG-3′. Hu TLR2, forward primer: 5′-TTAGCAACAGTGACCTAC-3′; reverse primer: 5′-GAACCAGGAAGACGATAA-3′. Hu TLR3, forward primer: 5′-TGTCTCATAATGGCTTGTC-3′; reverse primer: 5′-AGATTCCGAATGCTTGTG-3′. Hu TLR4, forward primer: 5′-GCTCACAATCTTATCCAATC-3′; reverse primer: 5′-GCCAGACCTTGAATACAA-3′. Hu TLR5, forward primer: 5′-CTAAGGTAGCCTACATTG-3′; reverse primer: 5′-TTCTGATAAGTGGATGAG-3′. Hu TLR6, forward primer: 5′-GGCAACTTATCACAACTG-3′; reverse primer: 5′-GATATGTTCACTTGGATAGC-3′. Hu TLR7, forward primer: 5′-AACCTCTCGCCATTACATAA-3′; reverse primer: 5′-GCCAACTTCACTTGAATCTC-3′. Hu TLR8, forward primer: 5′-CAATGCTCAAGTGTTAAGTG-3′; reverse primer: 5′-GTTACGCCTGCTATTCTG-3′. Hu TLR9, forward primer: 5′-TAAACCTGAGCTACAACA-3′; reverse primer: 5′-GTAATAACAGTTGCCGTC-3′. Hu β-actin, forward primer: 5′-GCGTGACATTAAGGAGAAG-3′; reverse primer: 5′-GAAGGAAGGCTGGAAGAG-3′. GAPDH, GAPDH, forward primer: 5′-CTGTGGGCAAGGTCATCCCTG-3′; reverse primer: 5′-AGACGGCAGGTCAGGTCCACC-3′.

### Short Interfering RNA and Plasmid Transfection

Human monocytes were transfected with short interfering RNA (siRNA) for control (si-Con), TLR4 siRNA (si-TLR4), or siRNA (si-IRF3) (Thermo Fisher Scientific, Waltham, MA, United States). The knockdown efficiency of siRNAs was determined one day post-transfection using western blotting.

### Western Blot

Treated cells were lysed in RIPA Buffer supplemented with a protease inhibitor cocktail (Thermo Fisher Scientific) and phosphatase inhibitor cocktail (Thermo Fisher Scientific). Equal amounts of protein were electrophoresed on a sodium dodecyl sulfate-polyacrylamide gel under reducing conditions followed by transfer to PVDF membranes. Blots were blocked with 5% BSA in TBST and western blots were probed with antibodies specific for CXCR3 (Abcam, ab154845, 1:1000), TLR4 (Novus, NB100-56566, 1:500, Centennial, CO, United States), pTBK1 (Cell Signaling, #5483, 1:1000, Danvers, MA, United States), TBK1 (Abcam, 1:1000), p-IRF3 (Cell Signaling, #4947S, 1:1000), IRF3 (Santa Cruz, sc-33641, 1:500), Histone H3 (Cell Signaling, #9715, 1:1000), Lamin B (Santa Cruz, sc-374015, 1:500), β-actin (Sigma-Aldrich, A5316, 1:5000). Secondary antibodies used were alkaline phosphatase conjugated to goat anti-mouse/rabbit IgG, or rabbit anti-Goat IgG (Jackson ImmunoResearch Labs, 1:10000). Signals were detected by SuperSignal West Dura Extended Duration or Pico PLUS Chemiluminescent Substrate (Thermo Fisher Scientific). Restore PLUS Western Blot Stripping Buffer was used to strip the membrane between two different target protein detection. All experiments had three or four biological replicates with representative blots presented in the figures.

### Monocyte Transmigration Assay *in vitro*

Boyden chambers (Corning Costar, NY, United States) were used to determine the transmigration of monocytes *in vitro*. Briefly, primary HBMECs were seeded (4 × 10^4^ cells/well) onto 6.5-mm polyester transwell inserts (3-μm pore size) and grown for 5days to achieve confluence. Human monocytes were pretreated with/without CXCR3 inhibitor AMG487 or TBK1 inhibitor Amlexanox for 1 h, followed by exposure of cells to HIV Tat protein for an additional 24 h and subsequently fluorescently labeled with 10 μM cell tracker green for 10 min at 37°C. Labeled cells (1 × 10^6^ cells/ml) were added to the upper compartment of transwell inserts in serum-free medium, and the chemokine CXCL10 was added to the basal side of the chamber at a final concentration of CXCL10 at 100 ng/ml. The transwell plates were incubated for 18 h at 37°C, followed by quantification of monocyte transmigration by measuring the number of migrated cells across the insert using a Synergy Mx fluorescence plate reader (BioTek Instruments, Winooski, VT, United States).

### Stereotaxic Injection

Eight-week-old C57BL/6 mice (male, *n* = 4) were microinjected with either saline or CXCL10 (100 ng/μl, 4 μl) into the brain using the microinjection parameters (coordinates +1.34 mm behind the bregma, +1.25 mm lateral from the sagittal midline at the depth of −4.0 mm to skull surface; flow rate: 0.5 μl/min) as previously described (Niu et al., 2019). After 24 h, animals were injected with BMMs isolated from CX3CR1-GFP mice at a concentration of 10^7^ cells/100 μl through the tail vein. Twenty-four hours after cell infusion, the animals were euthanized and subjected to transcranial perfusion with saline to remove CX3CR1-GFP+ monocytes from tissue blood vessels. Brain tissues were removed and frozen at −80°C until cryosection. Sections were then stained with anti-GFP (1:500; ab13970; Abcam), anti-F4/80 (1:100; ab6640; Abcam) and CXCR3 (1:50; sc-137140; Santa Cruz) antibodies and subjected to immunostaining as described above. Using four animals per group, different regions of the mouse brains were then quantified for triple-positive cells in the cortex, hippocampus, and thalamus regions.

### Statistical Analysis

Statistical analysis was performed using a two-tailed Student’s *t*-test for comparison of the two groups or one-way ANOVA with a Bonferroni’s *post hoc* test for multiple comparisons. For comparison between the two groups, an *F*-test was used to determine the equality of variances between groups. For comparison among multiple groups, a Brown–Forsythe test was used to determine the equality of variances among groups. In all cases, results were considered statistically significant when *p* < 0.05 for both Student’s *t*-test and one-way ANOVA test.

## Results

### HIV/SIV Infection Upregulates CXCL10/CXCR3 Axis and Enhances Macrophage Infiltration in the Brain

**FIGURE 1:**
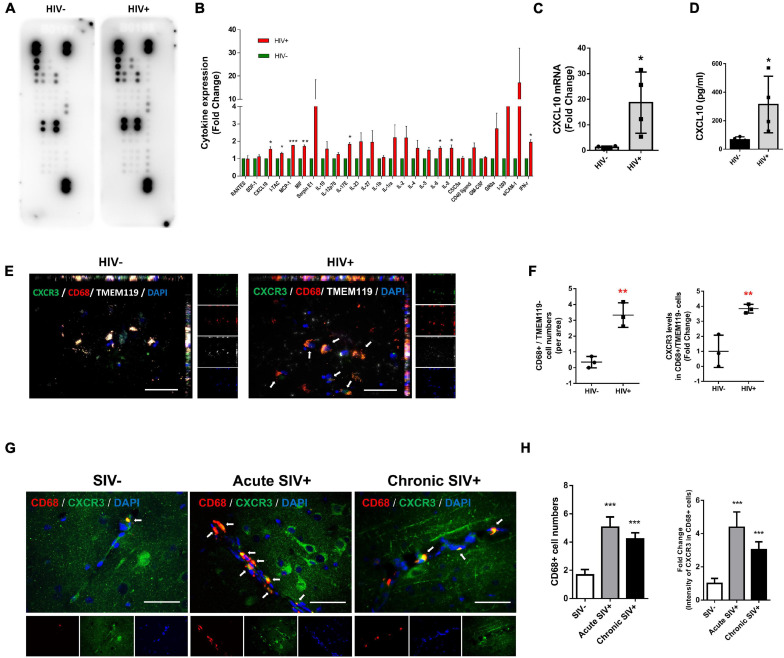
HIV/SIV infection upregulates CXCL10/CXCR3 axis and enhances macrophage infiltration in the brain. **(A)** Representative dot plots of Proteome Profiler Human Cytokine Array detecting the expression 26 cytokines/chemokines in the brain lysates of controls and HIV+ subjects. *N* = 3. **(B)** Quantification of cytokines/chemokines expression levels. **(C)** Real-time PCR analysis of CXCL10 mRNA expression in the brain lysates of controls and HIV+ subjects. *N* = 4. **(D)** ELISA analysis of CXCL10 protein expression levels in the brain lysates of controls and HIV+ subjects. *N* = 4. **(E)** Representative immunostaining of the frontal cortices of HIV-/HIV+ infected human (*N* = 3) stained with anti-CXCR3 (Green), anti-CD68 (macrophage marker, Red) and anti-TMEM119 (resident microglial marker, white) antibodies. Scale bar = 20 μm. Arrow: CXCR3+/CD68+/TMEM119– cells. **(F)** Quantification of CD68+/TMEM119– cells numbers and CXCR3 expression in CD68+/TMEM119– cells in the frontal cortices of HIV–/HIV+ infected human. Four or five images were analyzed per individual. Average values per individual were used for the statistical analysis by Student’s *t*-test. **(G)** Representative immunostaining of the frontal cortices of SIV–/acute SIV+/chronic SIV+ infected macaques stained with anti-CD68 (macrophage marker, Red) and anti-CXCR3 (Green) antibodies. Scale bar = 20 μm. Arrow: CD68+/CXCR3+ cells. **(H)** Quantification of number of CD68+ cells and CXCR3 expression levels in CD68+ cells in the frontal cortices of SIV-/acute SIV+/chronic SIV+ macaques. Four or five images were analyzed per animal. All data are presented as mean ± SD of four individual experiments (Biological replicates). *p < 0.05 versus HIV- group, **p < 0.01 versus HIV- group, ***p < 0.001 versus HIV– or SIV– group.

We first sought to screen the expression of cytokines/chemokines in the lysates of frontal cortices of HIV+ subjects and healthy controls. Cytokines/chemokines levels were determined semi-quantitatively using the Proteome Profiler Human Cytokine Array which detects 36 cytokines, chemokines, and acute-phase proteins. We were able to detect the expression of 26 cytokines/chemokines in the brain lysates of controls and HIV+ subjects (as shown in Figure 1A). As shown in Figure 1B, expression levels of CXCL10, I-TAC, MCP-1, MIF, IL17E, IL-6, IL-8, and IFN-γ were significantly upregulated in the frontal cortex lysates from HIV+ subjects compared with the healthy controls (CXCL10: 1.54-fold *p* = 0.0168, I-TAC: 1.31-fold *p* = 0.0374, MCP-1: 1.77-fold *p* = 0.000117, MIF: 1.70-fold *p* = 0.00138, IL17E: 1.54-fold *p* = 0.0168, IL-6: 1.61-fold *p* = 0.0262, IL-8: 1.61-fold *p* = 0.0301 and IFN-γ: 1.95-fold *p* = 0.0154). To validate our findings from the Proteome Profiler Human Cytokine Array, we performed expression of CXCL10 mRNA and protein levels in the same samples by real-time PCR and ELISA, respectively. As shown in Figures 1C,D, CXCL10 mRNA and proteins were significantly upregulated in the frontal cortices of HIV-infected patients compared with the healthy controls.

Based on the increased expression of CXCL10 in the frontal cortices of HIV-infected subjects, we next examined the influx of peripheral monocytes in the CNS in response to elevated CXCL10. The frontal cortices of postmortem brain tissues from HIV- and HIV+ individuals were assessed for expression of CXCR3, CD68 (macrophage marker)/TMEM119 (resident microglial marker) by immunofluorescence. As shown in Figures 1E,F, there were increased numbers of CD68+/TMEM119− cells in the brains of HIV+ individuals versus HIV- controls. Interestingly, the fluorescence intensity of CXCR3 was also increased in CD68+/TMEM119- cells in the brains of HIV+ subjects compared with HIV− controls.

Next, we validated the expression of CXCR3 and CD68 in the frontal cortices of control versus SIV infected macaques (both acute and chronic infection). As shown in Figures 1G,H, there were increased numbers of CD68+ positive cells as well as increased CXCR3 expression levels in CD68+ cells in the frontal cortices of both acute SIV+ and chronic SIV+ infected macaques compared with SIV− controls. There was no significant difference in either the numbers of CD68+ positive cells or the expression of CXCR3 in CD68+ cells in the frontal cortices of acute SIV+ versus Chronic SIV+ infected macaques. Although the TMEM119 marker could not be used in the experiments with Macaques, the results are in line with the human studies (Figures 1E–H).

### HIV Tat-Mediated Upregulation of CXCR3 in Human Monocytes

**FIGURE 2:**
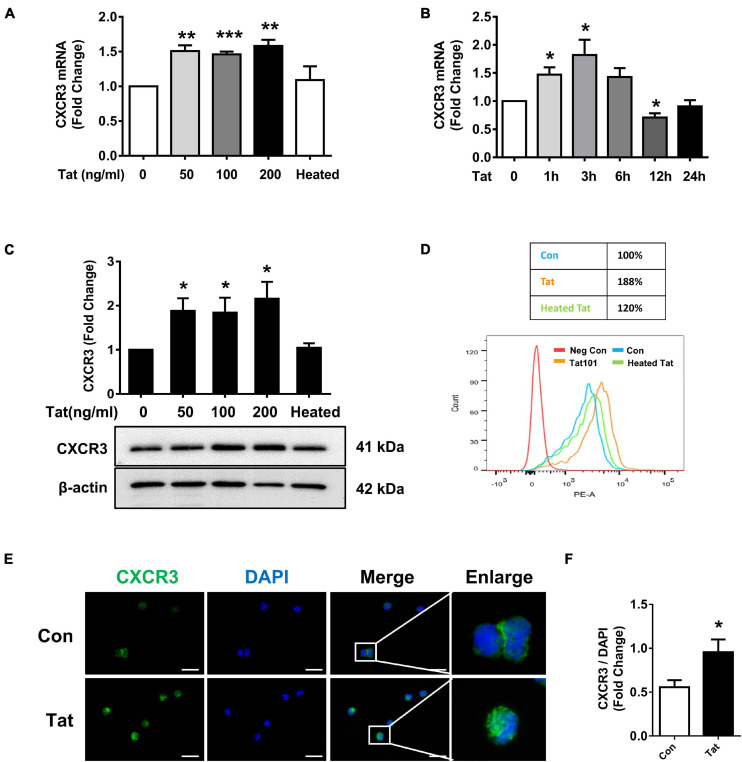
HIV Tat-mediated upregulation of CXCR3 in human monocytes. **(A)** Real-time PCR analysis of CXCR3 mRNA expression in human monocytes exposed to various concentrations of HIV Tat (50, 100, and 200 ng/ml) and heated HIV Tat (50 ng/ml). *N* = 3. **(B)** Real-time PCR analysis of CXCR3 mRNA expression in human monocytes exposed to HIV Tat (50 ng/ml) for varying time points. *N* = 3. **(C)** Representative Western blot and quantification of CXCR3 in the cell lysates from human monocytes exposed to various concentrations of HIV Tat (50, 100, and 200 ng/ml) and heated HIV Tat (50 ng/ml). *N* = 4. **(D)** Flow cytometry analysis of CXCR3 expression on the human monocytes exposed to HIV Tat and heated HIV Tat (50 ng/ml). **(E)** Representative images of human monocytes exposed to HIV Tat and stained with anti-CXCR3 antibodies. Scale bar 20 μm. *N* = 3. **(F)** Quantification of fluorescent intensities of CXCR3 staining in human monocytes (Student’s *t*-test). One-way ANOVA with *post hoc* test. All data are presented as mean ± SD of three or four individual experiments (Biological replicates). *p < 0.05 versus control group; **p < 0.01 versus control group; ***p < 0.001 versus control group.

Based on the findings that there is increased expression of CXCR3 that is associated with enhanced monocyte transmigration in the brains of HIV-infected patients and SIV+ infected macaques, we next sought to examine the effect of HIV Tat (as a surrogate of HIV infection) on the expression of CXCR3 in human monocytes *in vitro*. We first assessed dose- and time-course of CXCR3 expression following exposure of human monocytes to HIV Tat. For the dose-response curve, human monocytes were treated with varying concentrations (50, 100, and 200 ng/ml) of HIV Tat for 3 h, followed by assessment of CXCR3 mRNA by real-time PCR. As shown in Figure 2A, we observed an increase in CXCR3 mRNA expression in monocytes exposed to all the HIV Tat concentrations. Since even at 50 ng/ml of HIV Tat we observed upregulation of CXCR3, for ensuing studies this concentration of HIV Tat (50 ng/ml) was used, which is also a physiologically relevant concentration. As the level of HIV Tat protein in the CSF of infected individuals was shown to be ∼16 ng/ml (Westendorp et al., 1995), while that in the serum of HIV-positive patients, a concentration of HIV Tat of about ∼40 ng/ml or higher was demonstrated (Westendorp et al., 1995; Xiao et al., 2000; Flora et al., 2005; Rumbaugh et al., 2006; Eugenin et al., 2007). As expected, heated HIV Tat had no effect in inducing expression of CXCR3 (Figure 2A). For the time-course study, human monocytes were exposed to the chosen HIV Tat concentration (50 ng/ml) for various time periods (1–24 h) followed by assessment of CXCR3 mRNA by real-time PCR. In human monocytes exposed to 50 ng/ml HIV Tat, there was maximal induction of CXCR3 expression at 3 h (1.82-Fold, *p* = 0.0396, Figure 2B), following downregulation of mRNA expression at 12 h.

We next determined whether HIV Tat-mediated induction of CXCR3 also manifested as increased translation to protein levels. Human monocytes were exposed to HIV Tat at varying concentrations (50, 100, and 200 ng/ml) for 24 h followed by assessment of CXCR3 protein levels by western blotting. In human monocytes exposed to all the HIV Tat concentrations, there were upregulation of CXCR3 protein (Figure 2C). Additionally, we also assessed expression of CXCR3 protein by flow cytometry. As shown in Figure 2D, there was upregulation of CXCR3 receptors on the cell surface of human monocytes exposed to HIV Tat compared with control cells. These findings were also validated by immunofluorescence staining for CXCR3 in human monocytes cultured with or without HIV Tat. As shown in Figure 2E, we observed an increased fluorescent intensity of CXCR3 in monocytes exposed to HIV Tat compared with control cells. The quantification of CXCR3 fluorescence intensity is shown in Figure 2F.

### Involvement of TLR4 in HIV Tat-Mediated Upregulation of CXCR3 Expression

**FIGURE 3:**
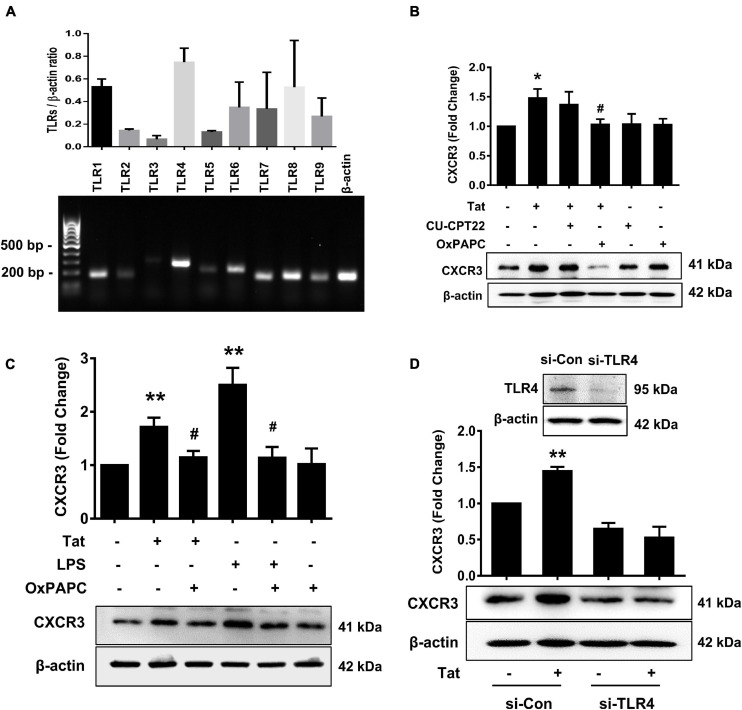
Involvement of TLR4 in HIV Tat-mediated upregulation of CXCR3 expression. **(A)** Comparative expression of TLR1 - TLR9 mRNA levels by RT-PCR analysis in human monocytes. **(B)** Representative Western blot and quantification of CXCR3 in the cell lysates from human monocytes pretreated with selective TLR1/2 inhibitor CU CPT22 (1 μM) and TLR2/4 inhibitor OxPAPC (30 μg/ml), followed by HIV Tat exposure for 24 h. *N* = 4. **(C)** Representative Western blot and quantification of CXCR3 in the cell lysates from human monocytes pretreated with TLR2/4 inhibitor OxPAPC (30 μg/ml), followed by LPS (100 ng/ml) or HIV Tat exposure for 24 h. *N* = 4. **(D)** Representative Western blot of silencing of TLR4 in human monocytes transfected with TLR4 siRNA (si-TLR4). Representative Western blot and quantification of CXCR3 in the cell lysates from human monocytes transfected with TLR4 siRNA and nonsense siRNA transfected controls (si-Con) followed by HIV Tat exposure. One-way ANOVA with *post hoc* test. *N* = 3. All data are presented as mean ± SD of three or four individual experiments (Biological replicates). *p < 0.05 versus control group; **p < 0.01 versus control group; #*p* < 0.05 versus HIV Tat or LPS group.

Toll-like receptors are known to play key roles in the generation of innate immune responses in various neurodegenerative diseases such as Alzheimer’s disease (Gambuzza et al., 2014), Parkinson’s disease (Kouli et al., 2019) as well as HAND (Alvarez-Carbonell et al., 2017). HIV Tat has been shown to activate TLR4 signaling directly via physical interaction with TLR4-MD2 (Ben Haij et al., 2013). To determine the role of TLRs in HIV Tat-mediated upregulation of CXCR3 expression in human monocytes, we first sought to examine the endogenous expression of various TLRs (TLR1, TLR2, TLR3, TLR4, TLR5, TLR6, TLR7, TLR8, and TLR9) in human monocytes by RT-PCR. As shown in Figure 3A, human monocytes express all of the TLRs, with strong expression of TLR4. Next, we assessed the involvement of TLRs in HIV Tat-mediated upregulation of CXCR3. For this, human monocytes were pretreated with either TLR1/2 antagonist CU-CPT22 (2 μg/ml) or the TLR2/4 antagonist OxPAPC (30 μg/ml) for 1 h, followed by exposure of cells to HIV Tat for additional 24 h and assessed for the expression of CXCR3 protein levels by western blotting. As shown in Figure 3B, in cells pretreated with CU-CPT22, HIV Tat was able to induce the expression of CXCR3, demonstrating thereby that TLR1/2 likely was not involved in HIV Tat-mediated upregulation of CXCR3 in human monocytes. On the other hand, pretreatment of cells with OxPAPC resulted in significant amelioration of HIV Tat-mediated upregulation of CXCR3 in human monocytes, indicating thereby that TLR4 played a role in HIV Tat-mediated upregulation of CXCR3. As shown in Figure 3C, both HIV Tat and LPS (positive control) were able to induce the expression levels of CXCR3 protein in human monocytes, and, further that, pretreatment of cells with OxPAPC significantly ameliorated HIV-Tat and LPS mediated upregulation of CXCR3. To further validate the involvement of TLR4 in HIV Tat-mediated upregulation of CXCR3 expression, we used a genetic knockdown approach by transfecting human monocytes with TLR4 (si-TLR4) or control scrambled siRNA. Transfection of human monocytes with si-TLR4 resulted in efficient knockdown of TLR4 expression (Figure 3D). Furthermore, in cells transfected with si-TLR4, HIV Tat failed to upregulate the expression of CXCR3 compared with cells transfected with scrambled siRNA (si-Con). These findings thus underscored the role of TLR4 in HIV Tat-mediated induction of CXCR3 in human monocytes.

### Involvement of pTBK1 in HIV Tat-Mediated Upregulation of CXCR3 Expression

**FIGURE 4:**
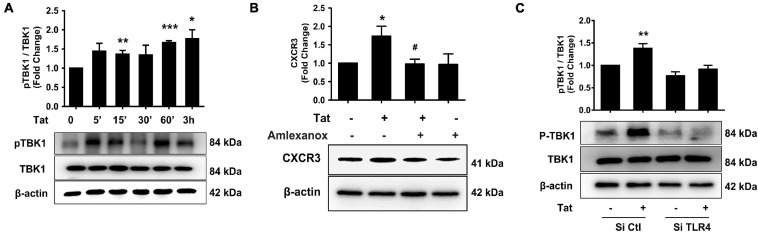
Involvement of pTBK1 in HIV Tat-mediated upregulation of CXCR3 expression. **(A)** Representative Western blot and quantification of pTBK1, TBK1 in the cell lysates from human monocytes exposed to HIV Tat for various time points (5 min – 3 h). *N* = 4. **(B)** Representative Western blot and quantification of CXCR3 in the cell lysates from human monocytes pretreated with TBK1 inhibitor Amlexanox (2 μM), followed by HIV Tat exposure for 24 h. *N* = 4. **(C)** Representative Western blot and quantification of pTBK1, TBK1 in the cell lysates from human monocytes transfected with TLR4 siRNA and nonsense siRNA transfected controls (si-Con) followed by HIV Tat exposure. *N* = 3. One-way ANOVA with *post hoc* test. All data are presented as mean ± SD of three or four individual experiments (Biological replicates). *p < 0.05 versus control group; **p < 0.01 versus control group; ***p < 0.001 versus control group; #*p* < 0.05 versus HIV Tat group.

To explore the mechanisms underlying HIV Tat-mediated induction of CXCR3, we examined the role of TLR4 and its downstream signaling pathway. It has been reported that following the activation of TLR3 or TLR4, there is phosphorylation and activation of TANK (TRAF family member-associated NF-κB activator)-binding kinase 1 (TBK1) (Bakshi et al., 2017). We next determined whether HIV Tat-mediated induction of CXCR3 in human monocytes involves activation of pTBK1. Human monocytes were exposed to HIV Tat (50 ng/ml) for various time periods (5 min to 3 h) followed by assessment of pTBK1 expression levels by western blotting. Exposure of human monocytes to HIV Tat resulted in a time-dependent increase in phosphorylation of TBK1, with activation as early as 15 min following HIV Tat exposure (Figure 4A). Since TBK1 activation occurred rapidly following the TLR4 activation and it controlled the downstream signaling of IRF3 phosphorylation (Clark et al., 2011), we chose 15 min post Tat treatment as the time in the following experiments. The specificity of TBK1 signaling pathway was subsequently assessed using a pharmacological approach-pretreatment of cells with the TBK1 inhibitor Amlexanox (2 μM). To examine the role of TBK1 in HIV Tat-mediated induction of CXCR3 expression, human monocytes were pretreated with Amlexanox for 1 h, followed by exposure of cells to HIV Tat for 24 h and assessment of CXCR3 protein expression by western blotting. As shown in Figure 4B, pretreatment of cells with Amlexanox, significantly inhibited HIV Tat-mediated induction of CXCR3 protein expression. Next, we sought to assess whether TBK1 pathway was downstream of TLR4. For this, human monocytes were transfected with either TLR4 siRNA or scrambled siRNA, followed by exposure of cells to HIV Tat for 15 min and assessed for phosphorylation of TBK1 by western blotting. In human monocytes transfected with si-TLR4, HIV Tat failed to induce the phosphorylation of TBK1 compared with cells transfected with scrambled siRNA (si-Con), wherein, as expected, HIV Tat exposure induced the expression of p-TBK1 (Figure 4C).

### HIV Tat-Mediated Phosphorylation of IRF3 and Its Nuclear Translocation

**FIGURE 5:**
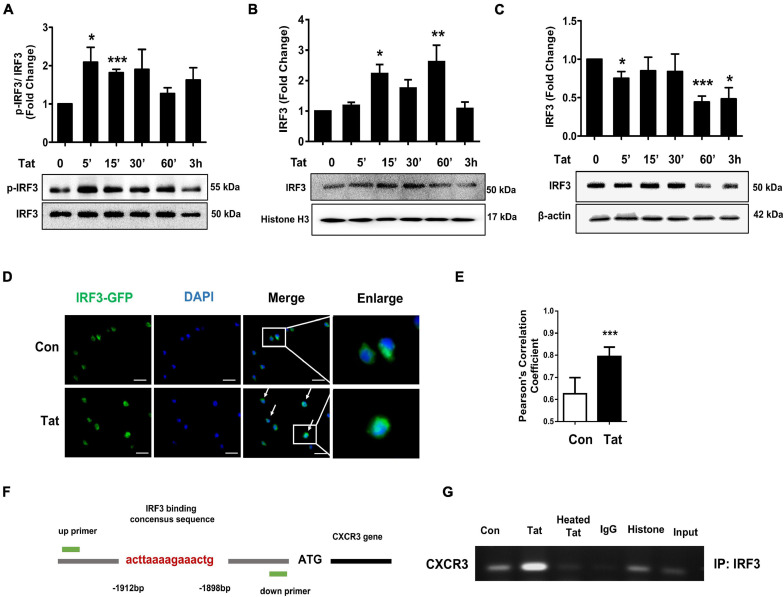
HIV Tat-mediated IRF3 phosphorylation and nuclear translocation. **(A)** Representative Western blot and quantification of p-IRF3, IRF3 in the cell lysates from human monocytes exposed to HIV Tat for various time points (5 min – 3 h). *N* = 3. **(B)** Representative Western blot and quantification of IRF3 in the nuclear cell lysates from human monocytes exposed to HIV Tat for various time points (5 min to 3 h). *N* = 4. **(C)** Representative Western blot and quantification of IRF3 in the cytoplasmic cell lysates from human monocytes exposed to HIV Tat for various time points (5 min to 3 h). *N* = 4. **(D)** Representative images of human monocytes transfected with IRF3-GFP plasmid, followed by HIV Tat exposure for 15 min. Scale bars: 20 μm. White arrow, IRF3 nuclear translocated cells. **(E)** Quantification of IRF3 nuclear translocation (Student’s *t*-test). *N* = 3. **(F)** Schematic illustration of IRF3 binding sequence on the promoter region of CXCR3. **(G)** ChIP assay demonstrating HIV Tat-mediated binding of IRF3 to the CXCR3 promoter. One-way ANOVA with *post hoc* test. All data are presented as mean ± SD of three or four individual experiments (Biological replicates). *p < 0.05 versus control group; **p < 0.01 versus control group; ***p < 0.001 versus control group.

Having determined that HIV Tat-mediated activation of TBK1 is involved in the induction of CXCR3, we next sought to investigate the transcriptional factor(s) that were involved in HIV Tat-mediated upregulation of CXCR3. Based on the report that TLR4-TBK1 activation can trigger phosphorylation and activation of interferon regulatory factor 3 (IRF3) (Bakshi et al., 2017), and since the promoter of CXCR3 has a consensus IRF3 binding site, we rationalized that IRF3 could likely bind to the CXCR3 promoter and regulate its transcription. To determine the involvement of the transcription factor IRF3 in HIV Tat-mediated upregulation of CXCR3, human monocytes were exposed to HIV Tat for various time points (5–180 min) and assessed for phosphorylation of IRF3 by western blotting. As shown in Figure 5A, in human monocytes exposed to HIV Tat, there was time-dependent phosphorylation of IRF3. Next, we assessed the nuclear translocation of IRF3 in human monocytes exposed to HIV Tat. For this, human monocytes exposed to HIV Tat for varying times were monitored for expression of IRF3 both in the nuclear and cytoplasmic fractions by western blotting. As shown in Figures 5B,C, exposure of human monocytes to HIV Tat resulted in a time-dependent increase in nuclear translocation of IRF3 with a concomitant decrease in its expression in the cytosol. Additional confirmation of these findings was done by fluorescence imaging of the cells. For this, human monocytes were transfected with IRF3-GFP for 24 h, followed by exposure of cells to HIV Tat for additional 15 min and imaging the cells by fluorescence microscopy using a (63×/1.4) objective lens. As shown in Figure 5D, exposure of cells to HIV Tat resulted in a significant increase in the intensity of IRF3 fluorescence in the nucleus. Quantification of nuclear translocation of IRF3 is presented in Figure 5E. We next examined whether HIV Tat mediates IRF3 binding to the intronic region of CXCR3, using an IRF3 ChIP assay. Human monocytes were treated with either HIV Tat or heated HIV Tat (1 h) followed by RNA extraction and ChIP assay (Figure 5F). As shown in Figure 5G, exposure of human monocytes to HIV Tat resulted in enhanced binding of IRF3 to the CXCR3 intronic promoter.

### Role of IRF3 in HIV Tat-Mediated Upregulation of CXCR3 Expression

**FIGURE 6:**
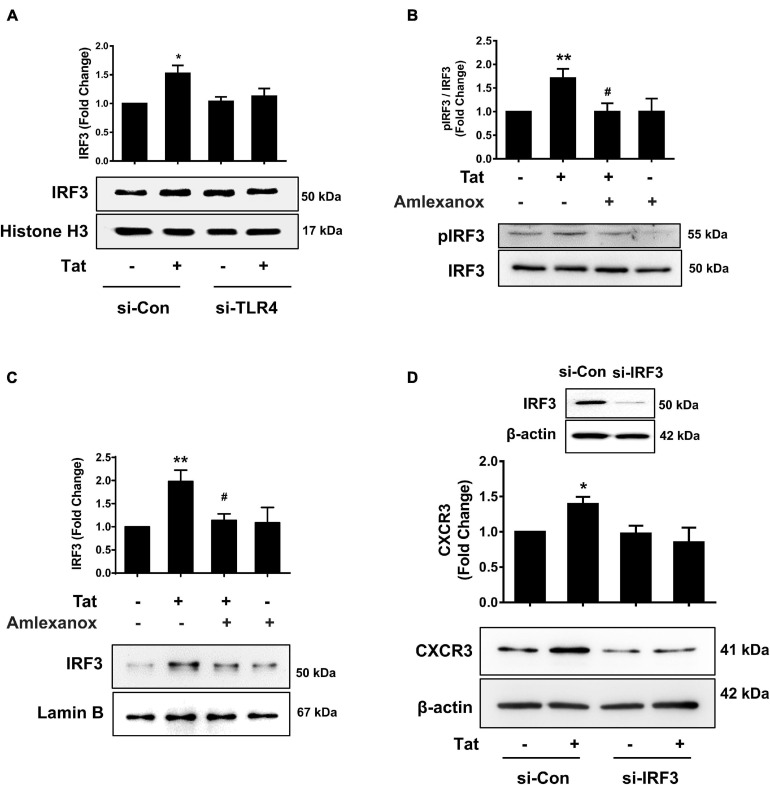
Involvement of IRF3 in HIV Tat-mediated upregulation of CXCR3 expression. **(A)** Representative Western blot and quantification of IRF3 in the nuclear cell lysates of human monocytes transfected with TLR4 siRNA and si-Con followed by HIV Tat exposure. *N* = 3. **(B)** Representative Western blot and quantification of p-IRF3, IRF3 in the cell lysates from human monocytes pretreated with TBK1 inhibitor Amlexanox (2 μM), followed by HIV Tat exposure for 15 min. *N* = 5. **(C)** Representative Western blot and quantification of IRF3 in the nuclear cell lysates of human monocytes pretreated with TBK1 inhibitor Amlexanox (2 μM), followed by HIV Tat exposure for 15 min. *N* = 3. **(D)** Representative Western blot of silencing of IRF3 in human monocytes transfected with IRF3 siRNA (si-IRF3). Representative Western blot and quantification of CXCR3 in the cell lysates from human monocytes transfected with IRF3 siRNA and nonsense siRNA transfected controls (si-Con) followed by HIV Tat exposure. *N* = 3. One-way ANOVA with *post hoc* test. All data are presented as mean ± SD of three or four individual experiments (Biological replicates). *p < 0.05 versus control group; **p < 0.01 versus control group; #*p* < 0.05 versus HIV Tat group.

To assess whether there is a possible linking activation of TLR4-pTBK1 signaling with activation of IRF3, human monocytes were transfected with either scrambled siRNA control or si-TLR4, followed by exposure of transfected cells to HIV Tat (15 min) and assessed for expression of IRF3 in the nuclear fractions. Excitingly, in human monocytes transfected with si-TLR4, HIV Tat exposure failed to increase the nuclear translocation of IRF3 compared with cells transfected with scrambled siRNA control, wherein HIV Tat exposure increased nuclear translocation of IRF3 (Figure 6A). To examine the role of pTBK1 in HIV Tat-mediated phosphorylation of IRF3, human monocytes were pretreated with TBK1 inhibitor Amlexanox (1 h), followed by exposure of cells to HIV Tat (additional 15 min) and assessing the cells for expression of phosphorylated and total IRF3. As shown in Figure 6B, pretreatment of human monocytes with Amlexanox resulted in abrogation of HIV Tat-mediated phosphorylation of IRF3. We also assessed the role of TBK1 in HIV Tat-mediated nuclear translocation of IRF3 in human monocytes. Briefly, the expression of IRF3 in nuclear fraction was assessed by western blotting in human monocytes pretreated with Amlexanox followed by HIV Tat exposure. In cells pretreated with Amlexanox, HIV Tat-mediated nuclear translocation of IRF3 was significantly ameliorated (Figure 6C). Having demonstrated that TLR4-pTBK1 signaling was involved in HIV Tat-mediated nuclear translocation of IRF3, we next sought to examine the role of IRF3 in HIV Tat-mediated upregulated expression of CXCR3. For this, human monocytes were transfected with either scrambled siRNA or si-IRF3, followed by exposure of cells to HIV Tat for 24 h, and assessed for expression of CXCR3 by western blotting. As shown in Figure 6D, transfection of human monocytes with IRF3 siRNA resulted in efficient knockdown of IRF3 expression. Furthermore, in cells transfected with si-IRF3, HIV Tat failed to induce the expression of CXCR3 compared with cells transfected with the scrambled siRNA control.

### HIV Tat-Mediated Upregulation of CXCR3 in Monocytes Leads to Increased Transmigration of These Cells Across the *in vitro* BBB Model

**FIGURE 7:**
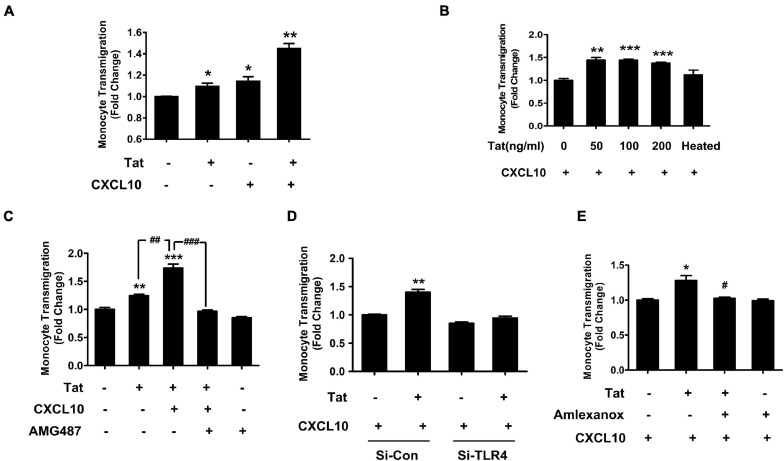
HIV Tat-mediated upregulation of CXCR3 in human monocytes facilitated its transmigration in an *in vitro* BBB model. **(A)** Human monocytes were pretreated with/without HIV Tat, followed by the transmigration assay in response to control media or CXCL10 (100 ng/ml). *N* = 3. **(B)** Human monocytes were pretreated with various concentrations of HIV Tat (50, 100, and 200 ng/ml), followed by the transmigration assay in response to CXCL10. *N* = 3. **(C)** Pretreatment of monocytes with the CXCR3 antagonist AMG487 ameliorated HIV Tat-induced monocyte transmigration in response to CXCL10. *N* = 3. **(D)** Human monocytes were transfected with TLR4 siRNA and nonsense siRNA transfected controls (si-Con) followed by exposure of transfected cells to HIV Tat and transmigration assay in response to CXCL10. *N* = 3. **(E)** Human monocytes were pretreated with TBK1 inhibitor Amlexanox followed by HIV Tat exposure and transmigration assay in response to CXCL10. *N* = 3. One-way ANOVA with *post hoc* test. All data are presented as mean ± SD of three or four individual experiments (Biological replicates). *p < 0.05 versus control group; **p < 0.01 versus control group; ***p < 0.001 versus control group; #*p* < 0.05 versus HIV Tat + CXCL10 group; ##*p* < 0.01 versus HIV Tat group; ###*p* < 0.001 versus HIV Tat + CXCL10 group.

We next determined whether HIV Tat-mediated upregulation of CXCR3 in human monocytes could contribute to increased monocyte transmigration, using an *in vitro* BBB model as described previously (Niu et al., 2019). Briefly, HBMECs were seeded onto a transwell until a confluent monolayer was formed. In parallel, human monocytes were pretreated with HIV Tat (50 ng/mL, for 24 h) and labeled with green fluorescence cell tracker. In the lower chamber of the transwell, exogenous CXCL10 (100 ng/ml) was added to aid in chemotaxis of the cells across the BBB. Subsequently, the green fluorescence cell tracker labeled monocytes were added onto the confluent HBMECs on the upper side of the transwell. There was increased transmigration of HIV Tat exposed monocytes across the BBB compared with control cells. A dramatic increase in monocyte transmigration was observed in monocytes pretreated with HIV Tat in the presence of CXCL10 (Figure 7A). Next, we sought to determine dose-dependent enhancement of monocyte transmigration across the BBB model (*in vitro* HBMECs culture) by HIV Tat. To address this, human monocytes were exposed to various concentrations of HIV Tat (50, 100, and 200 ng/ml) and assessed for transmigration across the *in vitro* BBB model in the presence of CXCL10. As shown in Figure 7B, all the concentrations of HIV Tat, we observed induction of monocyte transmigration, while heated HIV Tat failed to exert any effect.

We next sought to examine the specific role of CXCR3 in HIV Tat-mediated increase in monocyte transmigration. For this, human monocytes were pretreated with the CXCR3 antagonist AMG487 (1 μM) for 1 h, followed by exposure of cells to HIV Tat (24 h) and assessing the transmigration of these cells across an *in vitro* BBB model in presence or absence of CXCL10. While HIV Tat exposure enhanced monocyte transmigration even in the absence of CXCL10, there was a more dramatic increase in monocyte transmigration in the presence of CXCL10. Pretreatment of AMG487 resulted in significant amelioration of HIV Tat-mediated increase in monocyte transmigration in the presence of CXCL10 (Figure 7C).

To further validate the role of TLR4 in HIV Tat-mediated transmigration of monocytes in the presence of CXCL10, human monocytes were transfected with either TLR4 or scrambled siRNA for 24 h followed by exposure of cells to HIV Tat for additional 24 h, after which the cells were labeled with the cell tracker and added to the upper chamber of the transwell seeded with confluent monolayer of HBMECs. As shown in Figure 7D, in TLR4-siRNA transfected cells, HIV Tat failed to induce the transmigration of monocytes. In scrambled siRNA transfected cells, as expected, HIV Tat exposure resulted in significantly increased transmigration of monocytes across the BBB. Next, to determine the involvement of TBK1 in HIV Tat-mediated increase in monocyte transmigration, human monocytes were pretreated with Amlexanox and assessed for monocyte transmigration across the BBB. As shown in Figure 7E, pretreatment of monocytes with Amlexanox resulted in significant amelioration of HIV Tat-mediated increase in monocytes transmigration across the BBB.

### HIV Tat-Mediated Upregulation of CXCR3 Facilitated Monocyte Transmigration in Mice

**FIGURE 8:**
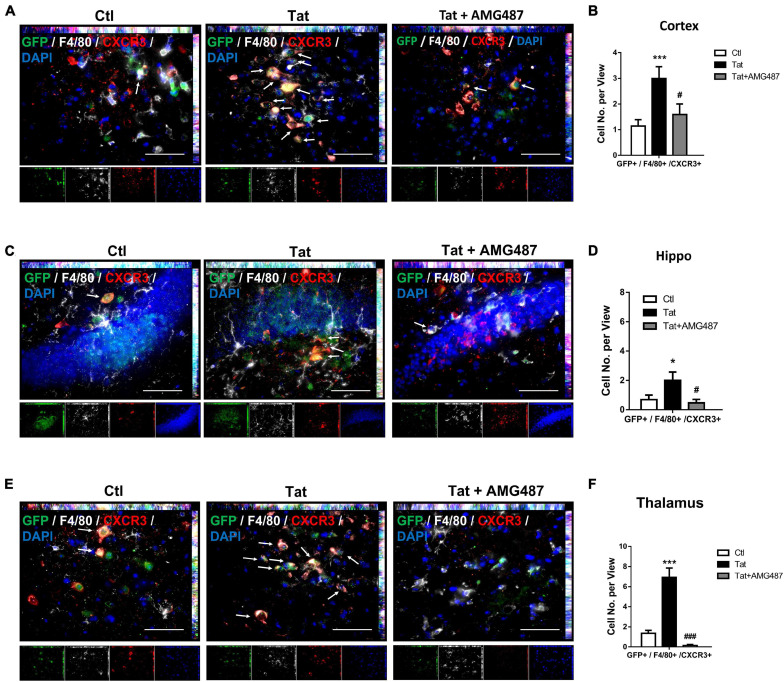
HIV Tat-mediated upregulation of CXCR3 facilitated monocyte transmigration in mice. **(A)** Representative images of CXCR3+/GFP+/F4/80+ cells in the cortex of mice administrated CXCL10. *n* = 4 per group. Arrow: CXCR3+/GFP+/F4/80+ cells; bar, 50 μm. **(B)** Quantification of CXCR3+/GFP+/F4/80+ cells in the cortex of mice administrated CXCL10. **(C)** Representative images of CXCR3+/GFP+/F4/80+ cells in the hippocampus of mice administrated CXCL10. *n* = 4 per group. Arrow: CXCR3+/GFP+/F4/80+ cells; bar, 50 μm. **(D)** Quantification of CXCR3+/GFP+/F4/80+ cells in the hippocampus of mice administrated CXCL10. **(E)** Representative images of CXCR3+/GFP+/F4/80+ cells in the thalamus of mice administrated CXCL10. *n* = 4 per group. Arrow: CXCR3+/GFP+/F4/80+ cells; bar, 50 μm. **(F)** Quantification of CXCR3+/GFP+/F4/80+ cells in the thalamus of mice administrated CXCL10. All data are presented as mean ± SD; five or six images per animal were analyzed. *p < 0.05 versus control group; ***p < 0.01 versus control group; #*p* < 0.05 versus HIV Tat group; ###*p* < 0.001 versus HIV Tat group.

Based on the premise that HIV Tat exposure resulted in increased expression of CXCR3 on monocytes, we next sought to determine the role of upregulated CXCR3 in mediating monocyte transmigration *in vivo*. For this, mouse bone marrow-derived monocytes (BMMs) were isolated from CX3CR1–GFP mice and pretreated with AMG487 for 1 h, followed by exposure of BMMs to HIV Tat for additional 24 h. To examine the monocyte migration, C57BL/6 mice were administered recombinant CXCL10 (100 μg/ml, 4 μl) stereotactically at the coordinates +1.34 mm behind the bregma, +1.25 mm lateral from the sagittal midline at the depth of −4.0 mm to skull surface, and 24 h later, treated or control BMMs from CX3CR1-GFP mice were injected via tail vein into the mice. Animals were euthanized 24 h later and brain sections were stained with anti-GFP, anti-CXCR3 and anti-F4/80 antibodies. The cortex, hippocampus and thalamus regions were assessed for distribution of GFP+/F4/80+/CXCR3+ cells. As shown in Figures 8A,B, there were increased numbers of GFP+/F4/80+/CXCR3+ cells in the cortices of mice administrated HIV Tat-exposed monocytes (CX3CR1-GFP) compared with control monocytes. In animals infused with BMMs isolated from CX3CR1-GFP mice that were pretreated with AMG487, HIV Tat exposure failed to induce the accumulation of GFP+/F4/80+/CXCR3+ cells in the cortex. Similar findings were also observed in the hippocampi and thalamus of mice (Figures 8C–F).

## Discussion

Myeloid cells such as the monocytes/macrophages are susceptible to HIV-1 infection and provide a reservoir for the virus as a means for immune evasion. These cells are also conduits for viruses spread into the CNS following their influx into the brain (Wong et al., 2019). Previous studies have demonstrated accumulation of perivascular macrophages around the perivascular cuffs in the CNS of subjects infected with HIV or SIV (Kim et al., 2003; Bokhari et al., 2011), thereby implicating the role of monocyte infiltration in the progression of HIV-1 infection in the CNS. While the effectiveness of cART in suppressing virus replication is well-documented (Ghosn et al., 2018; Jean et al., 2019), the caveat here is the lifelong dependence on cART, since interruption of therapy has been shown to reactivate the virus (Alvarez-Carbonell et al., 2017). Understanding the mechanism(s) by which these blood-borne monocytes influx into the brain could thus provide a basis for therapeutic strategies aimed at abrogating HIV-mediated neuroinflammation and the presence of viral reservoirs in the CNS.

The transmigration of monocytes into the CNS is a multistep process involving migration of these cells to the chemokine gradient in the brain, expression of adhesion molecules by these cells, regulation of these infiltrating cells by the endothelium as well as guidance by the pericytes (Proebstl et al., 2012; Gerhardt and Ley, 2015). Chemokines can be divided into four families based on the chemokine/ligand interaction motif to which they bind, including CC, CX3C, CXC, and XC (Čejková et al., 2015). Among these chemokines, RANTES, fractalkine, CCL2, SDF-1, and CXCL10 are primarily associated with monocyte transmigration. RANTES, also known as C-C motif chemokine ligand 5 (CCL5) belongs to the CC chemokine and binds to the CC-chemokine receptor 5 (CCR5), an important co-receptor for HIV uptake in cells (Karlsson et al., 2004). RANTES/CCR5 axis has been shown to contribute to the activation and interaction of monocytes with the endothelium, thus playing a critical role in the pathogenesis of atherosclerotic disease (von Hundelshausen et al., 2001). Similarly, fractalkine (CX3CL1, C-X3-C motif chemokine ligand 1), a member of the CX3C family with the unique receptor CX3C chemokine receptor 1 (CX3CR1), has been found to preferentially mediate arrest and migration of CD16+ monocytes and contribute to vascular and tissue injury during pathological conditions (Ancuta et al., 2003). Another chemokine stromal cell-derived factor 1/C-X-C motif chemokine ligand 12 (SDF-1/CXCL12), one of the CXC chemokines family that binds to the co-receptor CXC chemokine receptor 4 (CXCR4), is also a very important chemoattractant for both monocytes as well as resting T lymphocytes (Bleul et al., 1996). C-C motif chemokine ligand 2 (CCL2), also known as MCP-1, is a very widely studied chemokine and is found to enhance transmigration of HIV-infected leukocytes across the BBB (Eugenin et al., 2006). A study by the Berman group demonstrated that JAM-A, ALCAM and the chemokine receptor CCR2 was upregulated in mature HIV+ CD14+ CD16+ monocytes compared with HIV^exp^ CD14+ CD16+ monocytes (Veenstra et al., 2017). Moreover, exposure of astrocytes and endothelial cells to HIV Tat was also reported to increase the release of CCL2, with implications for increased monocyte transmigration (Park et al., 2001; Bozzelli et al., 2019). C-X-C motif chemokine ligand 10 (CXCL10)/IP-10 is a chemokine that has been shown to be dramatically upregulated in the plasma and brains of HIV-infected patients and SIV-infected rhesus macaques (Mehla et al., 2012). Based on these findings, CXCL10 has been speculated as a plasma inflammatory biomarker of immune activation in both viremic and aviremic HIV patients on cART therapy (Kamat et al., 2012) and, was found to be associated with a more rapid HIV/SIV neurological disease progression via the influx of CXCR3+ cells in the CNS (Ploquin et al., 2016).

Cytotoxic HIV Tat protein is a regulatory protein that is known to enhance virus transcription. Despite cART therapy, HIV Tat has been shown to persist in the CNS and hence has garnered immense attention in terms of its cytotoxic potential (Johnson et al., 2013). HIV Tat protein can be released from infected cells and promote the development and progression of HAND by affecting various CNS cells, including microglia, astrocytes and neurons. HIV Tat has been shown to directly cause synaptic loss and neuronal dysregulation (Kim et al., 2008; Santerre et al., 2019). While, HIV Tat protein can also promote neurotoxicity indirectly by inducing microglial activation and astrocyte dysfunction which, in turn, leads to increased production of pro-inflammatory cytokines in the CNS (Fan et al., 2011; Chivero et al., 2017; Thangaraj et al., 2018). Additionally, HIV Tat has also been shown to regulate monocyte transmigration by upregulating the expression on monocytes of adhesion molecules (Song et al., 2007; Duan et al., 2013) and also by inducing the release of proinflammatory cytokines from monocytes (Weiss et al., 1999; Pu et al., 2003). In the current study, we mainly focused on the effect of chemokine, CXCL10 and its cognate receptor in stimulating monocyte transmigration. Herein we demonstrate HIV Tat induced upregulation of the chemokine receptor CXCR3 on human monocytes. Our findings suggest that HIV Tat exposure mediates sequential activation of TLR4-pTBK-IRF3 signaling pathway in monocytes which, in turn, results in enhanced monocyte transmigration.

Toll-like receptor 4 (TLR4), is a member of TLR family that recognizes bacterial lipopolysaccharide (LPS) and plays a critical role in the pathogenesis of various neurodegenerative disease including Parkinson’s Disease (Campolo et al., 2019), Alzheimer’s disease (Walter et al., 2007), amyotrophic lateral sclerosis (Lee et al., 2015) and HIV-associated neuroinflammation (Hernandez et al., 2012). In a study by Ben Haij et al. (2015) it was shown that in myeloid cells HIV Tat can physically interact directly with TLR4-MD2 to upregulate the expression of proinflammatory cytokines such as IL-6/IL-8. Activation of TLR4 was also found to increase the migratory capacity of monocytes in response to CCL19 as well as VLA4-mediated monocyte migration (Totsuka et al., 2014). The role of TLR4 in HIV Tat-mediated upregulation of monocyte transmigration, however, has not been reported previously. Our findings implicate a critical role of TLR4 signaling pathway in HIV Tat-mediated transmigration of monocytes via upregulation of CXCR3.

Role of CXCR3 in virus latency and HIV-associated neuroinflammation has been reported previously (Khoury et al., 2016). CXCR3 is a chemokine receptor for chemokines CXCL9, CXCL10, CXCL11 and is expressed on peripheral monocytes (Vargas-Inchaustegui et al., 2010). CXCR3-dependent accumulation and activation of monocytes or macrophages have been shown to play a critical role in homeostatic arterial remodeling in response to hemodynamic stresses (Zhou et al., 2010) in chronic obstructive pulmonary disease (Costa et al., 2016), as well as in mycobacterial infection (Torraca et al., 2015). CXCL10, as the cognate chemokine of CXCR3 was found increased in serum of HIV infected patients on stable cART therapy compared with HIV− individuals (Ramirez et al., 2014). It has also been shown that elevated CXCL10 levels decreased T cell function and was strongly associated with HIV-1 viral loads (Gray et al., 2013; Ramirez et al., 2014). CXCL10 is a chemokine that is shown to be dramatically upregulated in response to HIV infection, and is considered as a biomarker in the clinical evolution of HIV infection (Kamat et al., 2012; Mhandire et al., 2017; Valverde-Villegas et al., 2018). CXCR3 is expressed on monocytes, Th1 lymphocytes, NK cells and NKT cells (Butler et al., 2017; Lei et al., 2019). While role of lymphocytes migrating to the CNS in response to CXCL10 has been well studied (Qin et al., 1998; Lunardi et al., 2015; Lei et al., 2019), the role of monocytes in this process remains less understood. For example, in HIV infected individuals, blood CXCR3-expressing memory CD4 T cells were found to be enriched in the proximity of cells containing inducible replication competent virus, likely recruited in response to the CXCL10 gradient (Banga et al., 2018).

The role of CXCR3 in monocyte influx into the CNS, however, has received less attention. Herein, for the first time, we demonstrate that exposure of monocytes to HIV Tat resulted in increased expression of CXCR3 on the surface of peripheral monocytes which, in turn, enhanced monocyte transmigration into the CNS. Moreover, we have also identified a novel molecular mechanism underlying HIV Tat-mediated upregulation of CXCR3 involving the TLR4 signaling pathway. We have also validated the binding of IRF3 to the CXCR3 promoter by ChIP assay.

**FIGURE 9:**
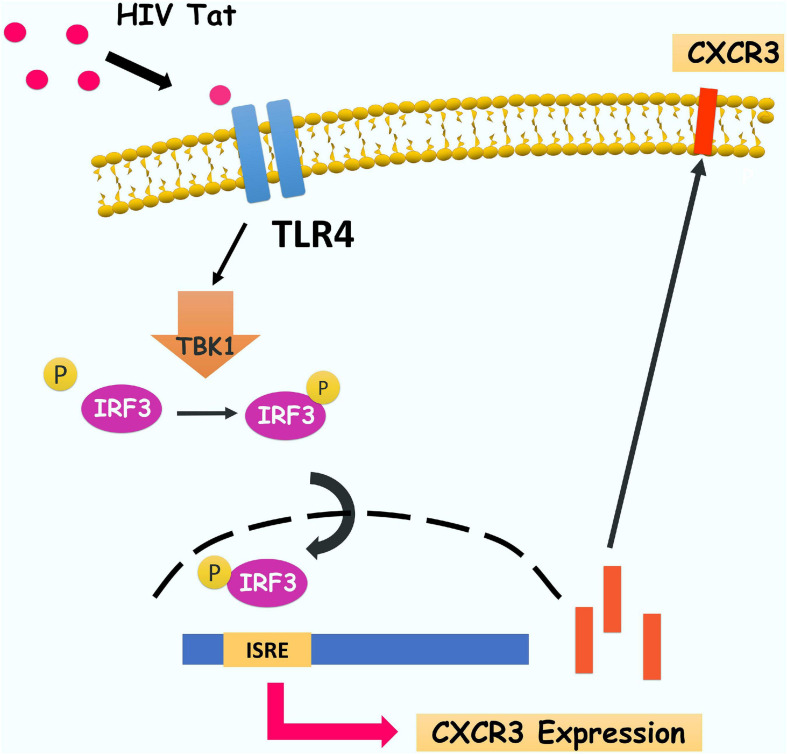
Schematic diagram demonstrating signaling pathways involved in HIV Tat-mediated induction of CXCR3 expression in human monocytes. HIV Tat-mediated TLR4 activation results in phosphorylation of TBK1 and subsequently induces phosphorylation and nuclear translocation of IRF3 which, leading, in turn, increases the binding of IRF3 and CXCR3 promoter and subsequently enhance CXCR3 expression. Increased CXCR3 ultimately leads to increased CXCL10-mediated monocyte transmigration into the brain.

In summary, our findings have delineated a detailed molecular pathway underlying HIV Tat-mediated upregulation of CXCR3 expression in human monocytes, leading to enhanced monocyte transmigration into the CNS (Figure 9). We have shown that HIV Tat induced the expression of CXCR3 in human monocytes involving activation of TLR4. Following this subsequent activation of TBK1 leads to phosphorylation and nuclear translocation of upregulated expression of CXCR3, in turn, facilitates increased transmigration of monocytes across the BBB, thereby promoting enhanced inflammation in the brain. Strategies aimed at blocking HIV Tat-mediated signaling pathways could thus be considered as future therapeutic option to dampen HIV Tat-mediated neuroinflammation, with ramifications also for other neurodegenerative disorders.

## Data Availability Statement

The raw data supporting the conclusions of this article will be made available by the authors, without undue reservation.

## Ethics Statement

The animal study was reviewed and approved by the UNMC Institutional Animal Care and Use Committee.

## Author Contributions

FN, KL, and SB designed the research. FN, KL, GH, and SM performed the research. FN, KL, SR, and SB wrote the manuscript. All authors contributed to the article and approved the submitted version.

## Conflict of Interest

The authors declare that the research was conducted in the absence of any commercial or financial relationships that could be construed as a potential conflict of interest.

## Publisher’s Note

All claims expressed in this article are solely those of the authors and do not necessarily represent those of their affiliated organizations, or those of the publisher, the editors and the reviewers. Any product that may be evaluated in this article, or claim that may be made by its manufacturer, is not guaranteed or endorsed by the publisher.
